# The relationship between obesity and diabetic nephropathy in China

**DOI:** 10.1186/1471-2369-14-69

**Published:** 2013-03-25

**Authors:** Hui-Mei Chen, Wen-Wen Shen, Yong-Chun Ge, Yi-De Zhang, Hong-Lang Xie, Zhi-Hong Liu

**Affiliations:** 1 Research Institute of Nephrology, Jinling Hospital, Nanjing University School of Medicine, Nanjing, 210002, China

**Keywords:** Diabetic nephropathy, ESRD, Obesity, Proteinuria

## Abstract

**Background:**

The epidemic of diabetic nephropathy (DN) has been paralleled by rapid increases in both obesity and diabetes in China. The aim of this study was to investigate the natural history of DN and the association of obesity and renal function with diabetes.

**Methods:**

In total, 264 patients with renal biopsy-confirmed DN were examined from 2002 to 2008 and followed up to June 2008 in our institute. Among these, 129 patients were classified into a Kidney Disease Outcomes Quality Initiative (K/DOQI) stage I subgroup. Weight status, clinico-histopathological features, the development of end-stage renal disease (ESRD) and increased proteinuria were evaluated at the baseline of biopsy and during the follow up. Lean, overweight and obese phenotypes were defined as body mass index (BMI) less than 25 kg/m^2^, 25–28 kg/m^2^, and more than 28 kg/m^2^ over, respectively.

**Results:**

In the patients with renal biopsy-confirmed DN, BMI was 25.5 ± 3.39 kg/m^2^, with 122 (46.2%), 83 (31.4%) and 59 (22.3%) having lean, overweight and obese phenotypes, respectively. Mean proteinuria was 3.09 ± 2.32 g/24 h, serum creatinine was 2.02 ± 2.02 mg/dL, and creatinine clearance rate (Ccr) was 96.0 ± 54.0 mL/min/1.73 m^2^. Compared with obese patients, lean patients had a lower Ccr, a higher percentage of anemia, more renal lesions and higher risk for ESRD (HR = 1.812, *P* = 0.048). The weight in obese patients decreased significantly after 27 months, and lean patients had a longer duration of diabetes than obese patients. Regarding patients at K/DOQI stage I, patients with DN showed similar duration of diabetes regardless of weight status. Minimal weight loss was recorded in obese patients during follow-up, and they exhibited greater glomerular hyperfiltration and higher risk for increased proteinuria (HR = 2.872, *P* = 0.014) than lean patients.

**Conclusions:**

In China, obesity is common in DN patients undergoing biopsy. Initial high levels of proteinuria and subsequent weight loss are the major characteristics of the natural course of DN. Obesity contributed to increased proteinuria at an early stage, while the lean phenotype was associated with ESRD development, especially at the later stages.

## Background

Obesity has become a serious worldwide problem. More than 300 million adults are classified as obese and the global number is predicted to reach 700 million by 2015. In the past, obesity was not a common condition among the population of China; however, this is no longer the case [1]. Obesity and diabetes have become a growing challenge, and one million new diabetic cases are reported in China every year. Increasing evidence suggests that obesity is a risk factor for diabetes and chronic kidney diseases. As a marker of obesity, high body mass index (BMI) has been reported to be related with diabetic nephropathy (DN) and end-stage renal disease (ESRD) [2]. A high prevalence of DN and ESRD has recently been demonstrated among Asians. During the past two decades, the prevalence of DN has dramatically increased in China [3,4], although the obesity profile among DN patients and the natural history of DN are not available in this region.

Obesity has been shown to aggravate renal injury associated with diabetes, although this effect is not consistent in previous studies. The evidence indicating a relationship between obesity and ESRD is paradoxical [5,6]. Tokashiki et al. showed that decreased BMI was the independent risk factor for chronic kidney disease, and the effect of obesity on renal survival varied with ethnicity [1,7]. Without intensive analysis, the possible role of obesity and weight loss on kidney function is still uncertain among Chinese patients with DN.

The first aim of our study was to explore the natural history of DN in Chinese patients. The second aim was to examine the impact of obesity on renal function in such a population. We screened patients presenting with type 2 diabetes, proteinuria, and biopsy-confirmed DN in our institute, and demonstrated an association of the obesity, weight loss with DN, and BMI with renal injury at different stages of diabetes. Obesity and overweight were defined according to the criteria outlined by the Working Group on Obesity of China [8]. The renal stages were evaluated by K/DOQI (Kidney Disease Outcomes Quality Initiative) guidelines.

## Methods

### Study population

Information on 15,141 cases was retrieved from patients, who underwent renal biopsy at a single unit during the period from July 2002 to June 2008 (Research Institute of Nephrology, Nanjing University School of Medicine, P.R. China). In this institute, the indications for renal biopsy included proteinuria and/or hematuria and/or renal insufficiency, with or without coexisting systemic disease [9]. The biopsies were performed by a group of clinicians, and renal biopsy specimens were examined by experienced nephrologists using techniques including light microscopy, immunohistology, and electron microscopy. Final diagnosis was made on the basis of clinical and histological features.

Written informed consent was obtained from all subjects prior to participation in the experimental protocol. The study was approved by the institutional review board of Nanjing University School of Medicine, and consistent with the Declaration of Helsinki. Initially, 757 patients with renal biopsy-confirmed DN were included, according to the following criteria: 1) type 2 diabetes mellitus (Standards of Medical Care in Diabetes, 2008, ADA [10]); 2) urinary protein excretion >400 mg/24 h; and 3) typical DN lesions in the glomeruli, tubulo-interstitium or blood vessels [11]. Patients with concomitant conditions such as IgA nephropathy and secondary renal damage due to hypertension were excluded [12,13]. All patients were recommended to follow-up in the out-patient center every 3 months; however, information was obtained for only 264 subjects to June 2008. The base characteristics of these 264 subjects were similar to those of the 757 subjects that were initially included (Additional file 1: Table S1).

**Table 1:** Epidemiological characteristics of subjects with diabetic nephropathy

	**Total**	**Lean Group BMI < 25**	**Overweight Group 25 ≤ BMI < 28**	**Obese Group BMI ≥ 28**	***P *value**
No. of patients	264	122	83	59	
BMI (kg/m^2^)	25.5 ± 3.39	22.6 ± 1.59	26.4 ± 0.85	31.3 ± 1.91	-
Age (years)	53.1 ± 9.06	54.1 ± 9.34	54.0 ± 8.97	53.1 ± 8.62	0.787
Male sex (%)	154(58.3%)	73(59.8%)	47(56.6%)	34(57.6%)	0.894
Known duration of diabetes (months)	111 ± 73.7	123 ± 74.8	110 ± 74.9	84.0 ± 62.1^**‡^	**0.002**
Known duration of proteinuria (months)	27.7 ± 41.7	23.7 ± 29.7	28.0 ± 39.7	27.8 ± 56.8	0.698
Fasting glucose (mg/dL)	124 ± 44	125 ± 48	117 ± 34	128 ± 51	0.338
HbA1c (%)	6.66 ± 1.33	6.81 ± 1.50	6.48 ± 1.20	6.61 ± 1.11	0.230
Mean blood pressure (mmHg)	108 ± 14.2	109 ± 14.5	105 ± 13.3	109 ± 14.8	0.139
Proteinuria (g/24 h)	3.09 ± 2.32	3.19 ± 2.19	2.97 ± 2.46	3.08 ± 2.69	0.821
≥3.5 g/24 h (%)	103 (37.4%)	48 (39.3%)	32 (38.6%)	23 (39.0%)	0.927
Serum albumin (g/dL)	3.45 ± 0.74	3.36 ± 0.69	3.46 ± 0.80	3.66 ± 0.74*	**0.017**
<3.5 g/dL (%)	141 (53.5%)	78 (63.9%)	37 (44.6%)**	26 (44.1%)**	**0.007**
Serum creatinine (mg/dL)	2.02 ±2.02	2.39 ± 2.27	1.52 ± 1.14**	2.00 ± 2.36	**0.010**
Ccr (mL/min/1.73 m^2^)	97.8 ± 53.4	81.6 ± 51.0	114 ± 57.6**	109 ± 64.8**	**< 0.001**
< 60 mL/min/1.73 m^2^ (%)	85 (32.2%)	52 (42.6%)	18 (21.7%)**	15 (25.4%)**	**< 0.001**
Uric acid (μmol/L)	400 ± 108	391 ± 110	401 ± 101	418 ± 113	0.340
Hemoglobin (g/dL)	11.2 ± 2.6	10.4 ± 2.3	11.7 ± 2.5**	12.3 ± 2.7**	**<0.001**
Anemia (%)	140 (54.9%)	84 (68.9%)	39 (47.0%) **	17 (34.7%) **	**< 0.001**
Volume of glomerulus (×10^6^ μm^3^)	4.97 ± 0.71	4.59 ± 0.64	5.13 ± 0.81	5.53 ± 0.68*	**0.031**
Mean global sclerosis (%)	27.5 ± 24.2	29.1 ± 25.9	26.8 ± 23.4	25.1 ± 21.7	0.634
Mesangial expansion (0–3)	2.51 ± 1.22	2.86 ± 1.14	2.33 ± 1.25*	2.06 ± 1.21**	**0.001**
K-W nodule (%)	145 (55.4%)	81 (66.4%)	40 (48.2%)*	24 (40.7%)**	**<0.001**
Interstitial fibrosis (0–3)	1.48 ± 0.72	1.57 ± 0.68	1.35 ± 0.72	1.48 ± 0.87	0.197
Tubular atrophy (0–3)	1.07 ± 0.21	1.14 ± 0.39	1.04 ± 0.37	0.97 ± 0.18*	**0.012**

NOTE. Values were expressed as expressed as mean ± SD and categorical data expressed as number (%). Abbreviation: BMI, body mass index; K-W, Kimmelstiel-Wilson; Ccr, creatinine clearance rate. To convert serum creatinine in μmol/L to mg/dL, multiply by 0.0113; creatinine clearance rate in mL/s/1.73 m^2^ to mL/min/1.73 m^2^, multiply by 60.0. The listed *P* values are based on ANOVA or Chi-square test. ^*^P < 0.05 and ^**^P < 0.01versus lean group. ^‡^*P* < 0.01 versus overweight group.

### Definitions

BMI was calculated as weight (kg) divided by height^2^ (m^2^). Based on the cut-off points in Chinese adults [8], BMI was classified as follows: obese, ≥28.0 kg/m^2^; overweight, 25.0-27.9 kg/m^2^; lean <25.0 kg/m^2^. The proteinuria per 24 hours and creatinine clearance rate (Ccr) were evaluated by 24-hour urine collection at the time of biopsy, and Ccr were calculated with the standard formula: urine creatinine (mg/dL) × urine volume (mL/min)/serum creatinine (mg/dL) and adjusted for body surface [14]. Other definitions were used: 1) nephrotic range proteinuria, 24-hour urine protein excretion ≥3.5 g; 2) hypoalbuminemia, serum albumin levels ≤3.5 g/L; 3) K/DOQ1 stage I, Ccr >90 mL/min/1.73 m^2^; and 3) renal insufficiency, Ccr <60 mL/min/1.73 m^2^. Insulin resistance was assessed using the Homeostasis Model Assessment of Insulin Resistance formula: fasting serum insulin (μU/mL) × fasting plasma glucose (mmol/L)/22.5 [15]. Anemia was defined as hemoglobin < 12 and < 11 g/dL in men and women, respectively.

Periodic acid-Schiff-stained paraffin-embedded sections at × 400 magnification were used to estimate pathologic lesions [16,17]. The percentages of globally sclerotic glomeruli and Kimmelstiel-Wilson (K-W) nodules were recorded from 50 glomerular sections for each patient. A semi-quantitative assessment of renal damage was performed according to mesangial proliferation, interstitial fibrosis, tubular atrophy and arteriolar hyalinosis [18].

The progression of renal injury was defined by the development of ESRD and increased proteinuria. The primary endpoint, ESRD, was defined as the requirement for permanent renal replacement therapy or serum creatinine exceeding 6.0 mg/dL for more than 1 month without other causes of renal dysfunction. The secondary endpoint, increased proteinuria, was defined as a 50% increase from the baseline level of proteinuria.

### Statistical analysis

Statistical analysis was performed using SPSS, version 11.0 (SPSS Inc, Chicago, IL). Quantitative variables were expressed as mean ± SD and compared by one-way ANOVA, followed with Least-Significant Difference (LSD) tests between each pair of groups. In the absence of a normal distribution, log-transformed data values were used for analysis and the Mann–Whitney test *U*-test was used when necessary. Qualitative variables were described as sample size (number of cases) and percentage (%), which were analyzed by chi-square test or Fisher’s exact test. Cumulative incidence of ESRD or disease progression was calculated using Kaplan-Meier survival probabilities (1- survival probabilities). Adjusted relative risks of mortality were calculated using Cox regression analysis. Two-tailed *P*-values less than 0.05 were considered statistically significant.

## Results

### Obesity profile of DN patients in China

**Figure 1:**
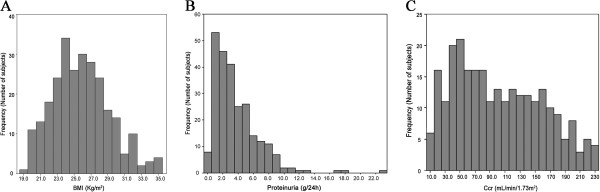
Histogram of weight mass index (A), proteinuria (B) and creatinine clearance rate (C) distribution among study subjects with diabetic nephropathy.

All 264 patients were diagnosed with type 2 diabetes and renal biopsy-confirmed DN. Analysis of the baseline characteristics are shown in Figure 1 and Table 1. The mean age of patients with DN was 53.1 ± 9.06 years (range 31–80). Of the DN patients, 58.3% were male. The average BMI was 25.5 ± 3.39 kg/m^2^ (range 18.9–36.6), and the frequency of BMI peaked at 24 kg/m^2^ (Figure 1A). Among these patients, 22.3% were obese, while 53.8% were obese/overweight. The average proteinuria was 3.09 ± 2.32 g/24 h (range 0.4–22.9), (Figure 1B), and 37.4% had nephrotic range proteinuria. Mean serum albumin was 34.5 ± 7.4 g/L, and 53.5% had hypoalbuminemia. Renal Ccr greatly varied in patients and the frequency of Ccr peaked around 50 mL/s/1.73 m^2^ (Figure 1C). Renal insufficiency occurred in 32.3% of subjects, 129 (48.9%) patients remained classified as early stage (K/DOQI stage I), and no patients suffered from ESRD. The mean serum creatinine was 2.02 ± 2.02 mg/dL.

Patients were divided into three groups on the basis of BMI as follows: obese, ≥28.0 kg/m^2^ (n = 59); overweight, 25–30 kg/m^2^ (n = 83); and lean, 18–25 kg/m^2^ (n = 122). The mean age and sex ratio among the three groups were similar, as were fasting glucose, HbA1c, mean blood pressure and previous therapy. At the time of diagnosis, the known duration of diabetes among patients ranged from a few months to 28 years. Compared with the obese patients, the lean patients suffered a longer duration of diabetes (*P* < 0.01), although the average history of proteinuria was similar among the three groups.

Among the lean patients, the percentage of renal insufficiency was 42.6%, presenting Ccr less than 60 mL/s/1.73 m^2^, which was significantly higher than that in the obese or overweight patients (*P* < 0.001, ANOVA). When compared with obese patients, the lean patients with DN showed higher levels of serum creatinine (*P* = 0.010), a higher percentage of hypoalbuminemia (*P* = 0.007) and anemia (*P* < 0.001), and greater mesangial expansion (*P* < 0.001), tubular atrophy (*P* = 0.012) and incidence of K-W nodules (*P* < 0.001). Obese patients had larger glomeruli than those with a lean phenotype (*P* = 0.031).

### Obesity and improved renal survival in DN patients

**Figure 2:**
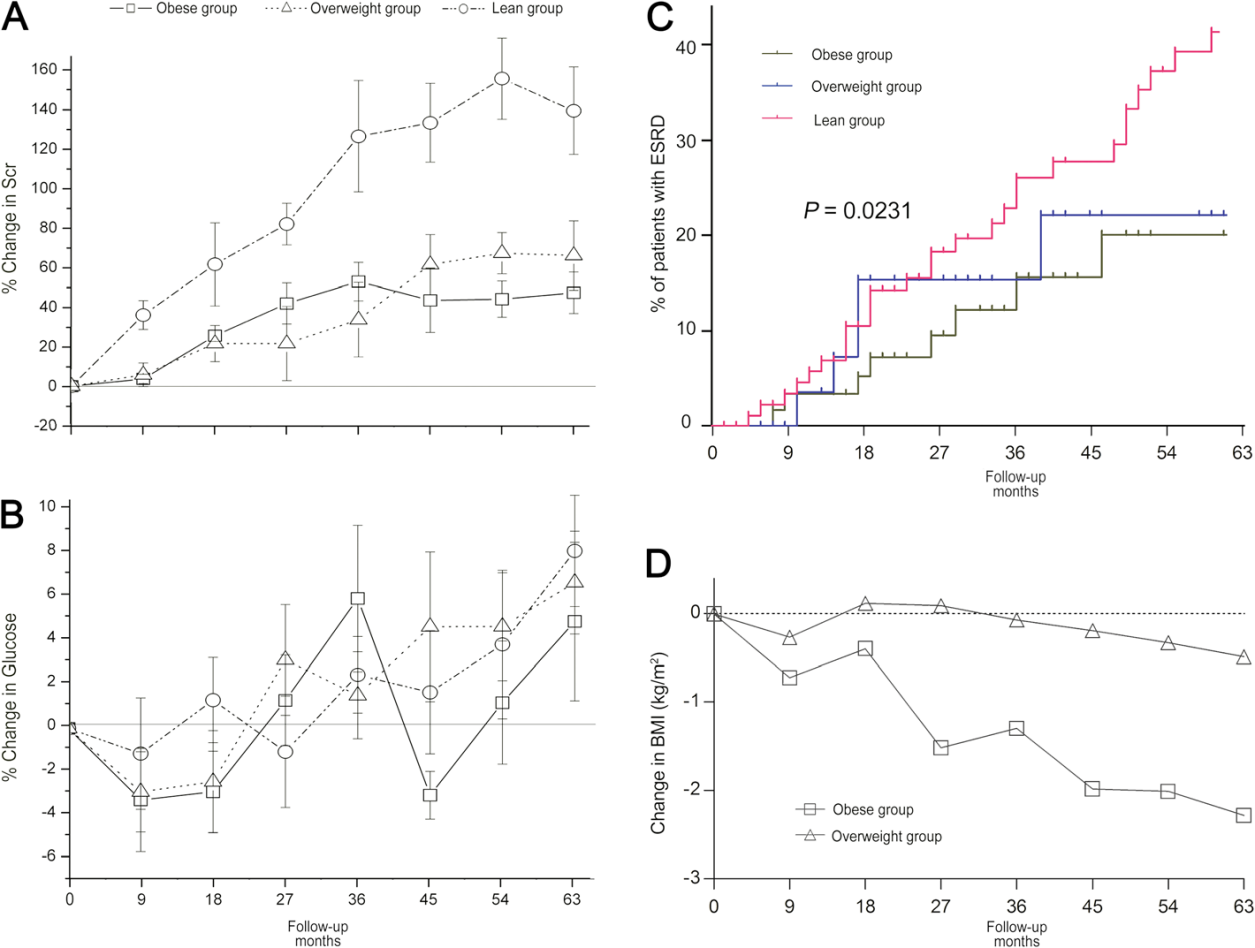
**The follow-up of glucose control and renal lesions in patients with DN (n = 264)**: **A, mean change in Scr (%); B, mean change in fasting glucose; C, cumulative incidence (%) of end-stage renal disease: patients with obesity have improved renal survival compared those with lean BMI (Kaplan–Meier analysis, log-rank, *P*** **= 0.0231); D, mean change in BMI (%).**

The information for follow-up was available for only 264 patients, and the median follow-up time was 39.0 months (range 0–87.0) (Figure 2). The median time of follow-up was 39.0, 36.0 and 36.0 months for the lean, overweight and obese groups, respectively. The levels of fasting glucose fluctuated but were similar among the three groups (Figure 2B). By the end of the study period, 74 cases (28.8%) had developed ESRD.

Compared with obese patients, the lean patients had a greater risk for developing ESRD (Figure 2). After 36 months of follow-up, the mean increment of serum creatinine was 126% in the lean group, while this was 52.7% and 33.5% in the overweight and obese groups, respectively (*P* < 0.001, ANOVA). By the end of the study period, 48 (39.4%) lean patients developed ESRD, and the relative risk of developing ESRD for the lean phenotype was 1.812 (95% CI 1.004–3.268; *P* = 0.048, Figure 2C). On the other hand, the mean BMI decreased among patients in the overweight or obese groups (Figure 2D), while no obvious change in BMI was detected in the lean group (data not shown). Regarding the obese patients, the reduction in BMI reached 1.30 kg/m^2^ and 2.28 kg/m^2^ after 36 and 63 months follow-up, respectively. The percentage of obesity in DN patients decreased from 22.3% at baseline to 19.3% at the end of the study period, while the rate of the lean phenotype increased from 46.2% to 58.3%.

### Obesity and increased proteinuria at early stage DN

Using the classification of chronic kidney disease, 129 subjects were classified as K/DOQI stage I and analyzed for an association between obesity and increased proteinuria at the early stage (Table 2). The demographic profile, including age and sex ratio, was similar between the total patient population studies and those at the early stage (data not shown). The patients at the early stage were further divided into three subgroups based on BMI: obese (n = 32), overweight (n = 51) and lean (n = 46).

**Table 2:** **The profile of sub-cohort with a creatinine clearance (Ccr) of more than 90 mL/min/1.73 m**^**2**^

	**Total**	**Lean sub-group**	**Overweight sub-Group**	**Obese sub-Group**	***P *value**
*N.* of patients	129	46	51	32	
Age (years)	52.6 ± 9.05	53.1 ± 9.36	52.1 ± 9.04	52.5 ± 8.82	0.883
Male sex (%)	78 (60.5%)	28 (60.9%)	31 (60.8%)	19 (59.3%)	0.987
BMI (kg/m^2^)	25.9 ± 3.05	23.0 ± 1.36	26.4 ± 0.84	30.5 ± 1.73	-
Ccr (mL/min/1.73 m^2^)	148 ± 38	138 ± 31	151 ± 40	159 ± 42**	**0.027**
>150 mL/min/1.73 m^2^ (%)	93 (72.1%)	28 (60.9%)	37 (72.5%)	28 (87.5%)*	**0.036**
Known duration of diabetes (months)	83.6 ± 68.8	91.7 ± 57.4	80.8 ± 63.8	76.6 ± 59.6	0.746
Known duration of proteinuria (months)	22.0 ± 25.6	20.0 ± 25.3	24.7 ± 26.1	20.5 ± 26.7	0.549
Fasting glucose (mg/dL)	131 ± 48	134 ± 52	122 ± 35	143 ± 60	0.184
HbA1c (%)	6.81 ± 1.46	7.04 ± 1.71	6.48 ± 1.21	7.03 ± 1.35	0.133
Mean blood pressure (mm Hg)	105 ± 13.4	106 ± 15.3	103 ± 11.7	105 ± 12.9	0.364
Proteinuria (g/24 h)	2.36 ± 2.33	2.39 ± 2.39	2.29 ± 2.32	2.43 ± 2.33	0.901
≥3.5 g/24 h (%)	30 (23.2%)	9 (19.6%)	13 (25.5%)	8 (25.0%)	0.760
Serum albumin (g/dL)	3.66 ± 0.77	3.47 ± 0.74	3.69 ± 0.77	3.78 ± 0.81	0.224
Serum creatinine (mg/dL)	0.88 ± 0.25	0.89 ± 0.21	0.89 ± 0.27	0.86 ± 0.24	0.987
Hemoglobin (g/dL)	12.7 ± 2.2	12.0 ± 2.0	12.8 ± 2.2	13.6 ± 2.2	**0.016**
Anemia (%)	33 (25.6%)	19 (41.3%)	10 (19.6%)	4 (12.5%)	**0.007**
High hemoglobin (%)	17 (13.2%)	5 (10.9%)	6 (11.8%)	6 (18.8%)	0.338
Volume of glomerulus (×10^6^ μm^3^)	5.45 ± 0.0.55	4.79 ± 0.31	5.44 ± 0.57*	6.40 ± 0.71**	**0.014**
Meanglobal sclerosis (%)	16.3 ± 16.4	13.3 ± 13.6	15.7 ± 17.0	21.7 ± 19.6	0.178
Mesangial expansion (0–3)	1.70 ± 0.80	1.96 ± 0.84	1.54 ± 0.78	1.62 ± 0.59	0.698
K-W nodule (%)	55 (42.6%)	27 (58.7%)	18 (35.3%)*	10 (31.3%)*	0.012
Interstitial fibrosis (0–3)	1.20 ± 0.66	1.62 ± 0.64	1.50 ± 0.59	1.23 ± 0.31	0.430
Tubular atrophy (0–3)	0.98 ± 0.30	1.05 ± 0.32	0.95 ±0.30	0.94 ± 0.24	0.267

NOTE. Values were expressed as mean ± SD or number (percentage); Abbreviation: BMI, body mass index; K-W, Kimmelstiel-Wilson; Ccr, creatinine clearance rate. To convert serum creatinine in mol/L to mg/dL, multiply by 0.0113; creatinine clearance rate in mL/s/1.73 m^2^ to mL/min/1.73 m^2^, multiply by 60.0. The listed *P* values are based on ANOVA or Chi-square test. ^*^P < 0.05 and ^**^P < 0.01 versus lean group.

Among the patients at the early stage, the known duration of diabetes was similar among the three subgroups, although this factor differed in the total patient population studied and those with DN. The known duration of proteinuria was similar among the three groups at the early stage, as were the levels of fasting glucose, HbA1c and mean blood pressure. At the early stage, greater renal damage was observed in obese patients compared with lean patients, with greater glomerular hyperfiltration and a lower percentage of anemia (*P* = 0.027 and *P* = 0.007, respectively).

**Figure 3:**
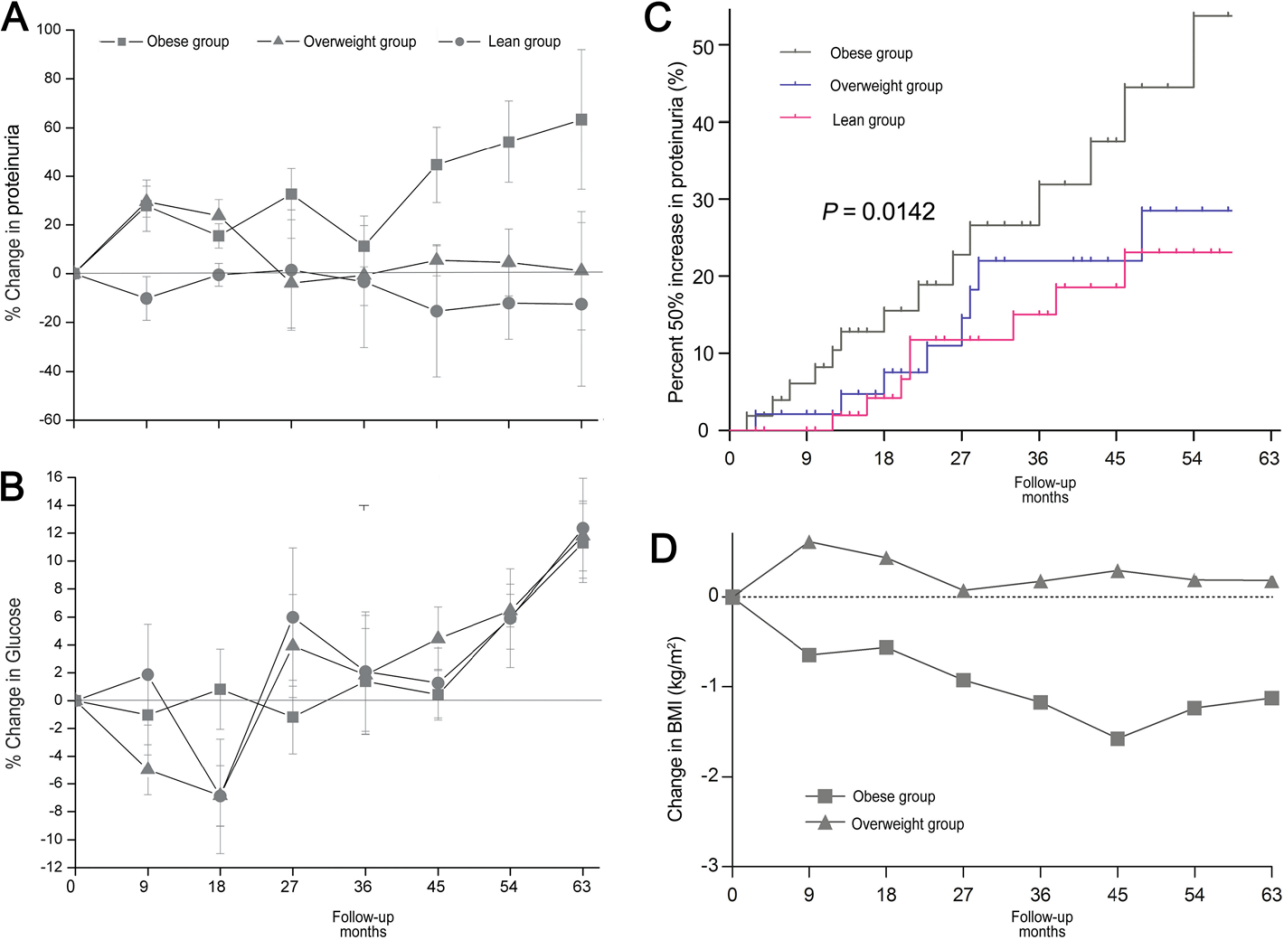
**The follow-up of glucose control and renal lesions in subjects at K/DOQI stage I (n = 129). A**, mean change in proteinuria (%); **B**, mean change in fasting glucose; **C**, cumulative incidence (%) of proteinuria progression: patients with obesity have higher risk of progress of proteinuria compared those with lean BMI (Kaplan–Meier analysis, log-rank, *P* = 0.0142); **D**, mean change in BMI (%).

Among the patients at the early stage, the median time of follow-up was 30.0 months (Figure 3). The fasting glucose levels were almost stable during follow-up (Figure 3B). By the end of the study period, only 2 (1.55%) cases developed ESRD, while 30 (24.8%) patients had increased proteinuria. The mean levels of proteinuria increased in the obese patients, reaching 11.3% and 63.3% after 36 and 63 months, respectively (Figure 3A). However, no obvious increase in proteinuria was detected in the lean and overweight subgroups. The relative risk for increased proteinuria was high in the obese patients, and 12 (37.5%) exhibited a 50% increase in proteinuria compared with the baseline levels. Using the lean group as a reference, obesity showed a significantly higher risk for increased proteinuria (Hazard ratio 2.872 [95% CI 1.239–6.659], *P* = 0.014). Compared with the total population of obese patients studied, the decrease in mean BMI was relatively small in obese patients at the early stage (*P* = 0.018) and was only 1.12 kg/m^2^ after 63 months of follow-up (Figure 3D). The overweight patients at the early stage did not show obvious changes in BMI.

## Discussion

With rapid nutrition and lifestyle transitions over the past two decades, the global rate of obesity has been increasing at an alarming rate, including in China [19]. The frequency of diabetes has risen in tandem, as obesity is a component of risk factors for diabetes [20]. Today, China has the largest number of patients with obesity and diabetes mellitus in the world. Furthermore, increasing numbers of studies have investigated the relationship between obesity and DN in this ethnic group. This study shows for the first time that obesity and weight loss are common in Chinese patients with biopsy-confirmed DN.

Although all the patients were from a single unit, the profile of patients is representative of the characteristics of Chinese patients with renal disease, especially those in Eastern China. In our institute, there was an abrupt rise in the number of patients undergoing biopsy after 1985, reaching 500 cases per year in 1991, with a further increase to over 1,000 cases per year after 1998 [9]. Recently, the number of renal biopsies performed each year has exceeded 2,000. The expanded pool of renal biopsies from kidney disease patients makes the epidemiologic investigation more reliable. However, the patients were not regularly interviewed in our institute because of concerns of economy and/or distance. The follow-up information, therefore, was relatively limited. Among all 757 patients with DN, follow-up information was available for only 264 subjects. The baseline characteristics were similar between the 757 patients and 264 patients (data not shown).

In Chinese patients with biopsy-confirmed DN, 22.3% presented with obesity and 53.7% with overweight/obesity. The prevalence of obesity was as high as 24.8% in patients with early stage DN. Compared with the prevalence in the general Chinese population [21], obesity was more common in the patients with DN. High prevalence of obesity has been confirmed in other ethnic groups [22]. The obese patients with DN in this study exhibited weight loss with diabetes during follow-up. On average, BMI decreased by 4.62% (2.28 kg/m^2^) after 63-months in obese patients, while only moderate decreases were observed in patients at the early stage. This suggested that weight loss was a predictive marker of poor or declining health for diabetic patients, which was consistent with the findings of other studies [23]. Accordingly, the patients with lean phenotype had a longer duration of diabetes than those with obesity. Compared with obese patients, lean patients exhibited more severe renal injury, including higher percentage of renal insufficiency and a higher score for tubular lesions. Taking the lean group as the reference, obesity and overweight significantly decreased the risk for ESRD. This confirmed that a proportion of lean patients suffer from both DN development and weight loss before renal biopsy, and indicates that weight loss is the major characteristics of the natural course of DN and shows a predictive effect on DN in China. Thus, the lean phenotype indicates disease progression. However, a protective role for obesity in renal disease progression was not identified in this retrospective study.

On the other hand, weight control was generally thought to decrease the risk for renal injury associated with obesity and diabetes. Our previous study also proved that weight loss decreased urinary protein by 51% after 24 months in subjects with obesity-related glomerulopathy (ORG) [24]. Saiki et al. reported the protective effect of weight loss using a formula diet on renal function in obese patients with DN [25]. These results contrast with our findings. In fact, more studies have suggested the “obesity paradox” in diabetes and renal diseases [26]. In the present study, a lean phenotype and weight loss were associated with a “deterioration phenomenon” and progression of renal injury in DN patients in China. On the other hand, obesity contributed to increased proteinuria at the early stage of DN. Among patients at CKD stage I, obese patients showed greater hyperfiltration than lean patients. Obesity further increased the risk for increased proteinuria by 2.872 compared with the lean patients. Similar results were found in the subjects with normal renal function or at the early stage of disease. Thus, the present study using one population of DN patients showed a varied effect of obesity and weight loss on the progression of DN.

Much evidence has shown that the different effect of weight loss resulted from two different mechanisms of weight loss: intentional and unintentional. In brief, diet control and extra exercise leads to intentional weight loss, mainly from the fat mass, thus improving insulin resistance, inflammation, and subsequent renal injury. However, with the progression of diabetes, high sugar levels lead to dehydration, muscle breakdown and an unintentional weight loss, which predicted clinical deterioration. Zoccali [27] suggested that maintenance of the current weight was the best way to protect against DN. Unfortunately, BMI was the only marker used to evaluate obesity in the present study, and it was difficult to classify the source of weight loss. As a retrospective study, the different effect of weight loss on DN was suggested by the “obesity paradox” with no direct evidence in support of this phenomenon. Further studies, especially prospective cohort studies, are required to compare the effect of intentional and unintentional weight loss on DN, which might help to explain the “obesity paradox” and “weight loss paradox” in China.

It should be pointed out that there are several additional limitations associated with this retrospective study. Serum creatinine levels were used in the present study to define ESRD, although this is not a sufficient measure of the loss of renal function, as it can be influenced by muscle mass, nutritional status and gender. In our institute, patients are usually diagnosed with ESRD, when serum creatinine levels exceed 6 mg/dL for more than 1 month, and other causes of renal disease are excluded. The value of “6 mg/dL” was adopted as a “cut-off” based on the value commonly used in Chinese medical schools [28]. Although insufficient, serum creatinine is a conveniently tested measure in clinical practice, and was therefore used as a marker of the loss of renal function.

Because of the limitations of this study in terms of population number and follow-up information, the development of ESRD does not reflect the progression of renal injury in all patients with DN. According to the different features of DN at different stages, two end-points were used to assess the progression of DN in the present study. The development of ESRD was used as the first endpoint, and increased proteinuria was used as the second endpoint only for those patients at the early stage. It is well known that early stage patients exhibit micro-albuminuria or moderate proteinuria, with normal levels of serum creatinine [10]. The levels of albuminuria/proteinuria were shown to be associated with the severity and out-come of renal injury. At a later stage, most patients had gross proteinuria and increased serum creatinine. High levels of serum creatinine are linked with ESRD, while the levels of proteinuria fluctuate with diet and other factors. Using diverse end-points, our study shows that obesity is significantly associated with increased proteinuria in early stage patients, and the lean phenotype is linked with ESRD in all DN patients.

It should also be noted that a direct effect of obesity has been shown on renal function contributing to proteinuria and renal lesions [29]. Some obese patients probably suffered from ORG prior to the diagnosis of type 2 diabetes or DN. It is difficult to distinguish between these two diseases based on natural history, or from histological features. ORG is characterized by glomerulomegaly and focal segmental glomerulosclerosis, which can also be observed in DN. Patients with DN, especially obese subjects, might present with ORG simultaneously. Previous ORG and/simultaneous ORG partly added to the difference in progression of CKD between lean and overweight/obese patients.

## Conclusions

Overall, our study demonstrates that obesity is very common among DN patients undergoing renal biopsy in China, which is associated with the most rapid increases in both obesity and diabetes globally. Obese patients showed a natural course of DN – heavy proteinuria initially, followed by weight loss. Obesity seemed to be associated with increased proteinuria at the early stage, while it was associated with a benefit in terms of improved renal survival at the later stages. Weight loss was accompanied by the progression of diabetic nephropathy with a prolonged diabetic duration. Lean patients exhibited a greater acceleration of disease toward ESRD than obese patients. The present study further elucidates the mechanism of progression of DN in China in relation to obesity and warns of the concurrence of weight loss and the development of diabetes. Regular evaluation of weight is important for the patients with DN, as well monitoring of glucose levels, proteinuria and serum creatinine.

## Competing interests

The authors declare that they have no competing interest.

## Authors’ contribution

CHM and LZH designed the study. SWW, GYC and ZYD collected samples and clinical information. XHL performed the laboratory assays. CHM performed the statistical analyses and wrote the manuscript. The final version of the manuscript was approved by all authors.

## Pre-publication history

The pre-publication history for this paper can be accessed here:

http://www.biomedcentral.com/1471-2369/14/69/prepub

## Supplementary Material

Additional file 1: Table S1 Epidemiological characterisstics of subjects with diabetic nephropathy. NOTE. Values were expressed as expressed as mean ± SD and categorical data expressed as number (%). Abbreviation: BMI, body mass index. To convert serum creatinine in μmol/L to mg/dL, multiply by 0.0113; creatinine clearance rate in mL/s/1.73 m^2^ to mL/min/1.73 m^2^, multiply by 60.0. The listed *P* values are based on student’s test or Chi-square test. ^*^P < 0.05 and ^**^P < 0.01versus lean group. ^‡^*P* < 0.01 versus overweight group. Click here for file

## References

[B1] Ji CY Cheng TO Epidemic increase in overweight and obesity in Chinese children from 1985 to 2005 Int J Cardiol 2009 132 1 1 10 10.1016/j.ijcard.2008.07.003 18835050

[B2] Hsu CY McCulloch CE Iribarren C Darbinian J Go AS Body mass index and risk for end-stage renal disease Ann Intern Med 2006 144 1 21 28 16389251 10.7326/0003-4819-144-1-200601030-00006

[B3] Hsieh MC Hsieh YT Cho TJ Chen JF Lin SD Chen HC Tu ST Remission of diabetic nephropathy in type 2 diabetic Asian population: role of tight glucose and blood pressure control Eur J Clin Invest 2011 41 8 870 878 10.1111/j.1365-2362.2011.02479.x 21299554

[B4] Luk A Chan JC Diabetic nephropathy–what are the unmet needs? Diabetes Res Clin Pract 2008 82 Suppl 1 S15 S20 18952313 10.1016/j.diabres.2008.09.033

[B5] Speakman JR Westerterp KR Reverse epidemiology, obesity and mortality in chronic kidney disease: modelling mortality expectations using energetics Blood Purif 2010 29 2 150 157 10.1159/000245642 20093821

[B6] Valocikova I Valocik G Kristofova B Druzbacka L Obesity paradox and chronic kidney disease Bratisl Lek Listy 2011 112 7 402 406 21744737

[B7] Pei YP Greenwood CM Chery AL Wu GG Racial differences in survival of patients on dialysis Kidney Int 2000 58 3 1293 1299 10.1046/j.1523-1755.2000.00285.x 10972693

[B8] Zhou BF Predictive values of body mass index and waist circumference for risk factors of certain related diseases in Chinese adults–study on optimal cut-off points of body mass index and waist circumference in Chinese adults Biomed Environ Sci 2002 15 1 83 96 12046553

[B9] Li LS Liu ZH Epidemiologic data of renal diseases from a single unit in China: analysis based on 13,519 renal biopsies Kidney Int 2004 66 3 920 923 10.1111/j.1523-1755.2004.00837.x 15327382

[B10] Alberti KG Zimmet PZ Definition, diagnosis and classification of diabetes mellitus and its complications. Part 1: diagnosis and classification of diabetes mellitus provisional report of a WHO consultation Diabet Med 1998 15 7 539 553 10.1002/(SICI)1096-9136(199807)15:7<539::AID-DIA668>3.0.CO;2-S 9686693

[B11] Jennette JLO JC Schwartz MM Silva FG Heptinstall’s Pathology of the Kidney 1998 5 Lippincott Willians & Wirkins

[B12] Huang F Yang Q Chen L Tang S Liu W Yu X Renal pathological change in patients with type 2 diabetes is not always diabetic nephropathy: a report of 52 cases Clin Nephrol 2007 67 5 293 297 17542338 10.5414/cnp67293

[B13] Tone A Shikata K Matsuda M Usui H Okada S Ogawa D Wada J Makino H Clinical features of non-diabetic renal diseases in patients with type 2 diabetes Diabetes Res Clin Pract 2005 69 3 237 242 10.1016/j.diabres.2005.02.009 16098920

[B14] Lemann J Bidani AK Bain RP Lewis EJ Rohde RD Use of the serum creatinine to estimate glomerular filtration rate in health and early diabetic nephropathy. Collaborative study group of angiotensin converting enzyme inhibition in diabetic nephropathy Am J Kidney Dis 1990 16 3 236 243 2399915 10.1016/s0272-6386(12)81023-7

[B15] Chen HM Chen Y Zhang YD Zhang PP Chen HP Wang QW Li LS Liu ZH Evaluation of metabolic risk marker in obesity-related glomerulopathy J Ren Nutr 2011 21 4 309 315 10.1053/j.jrn.2010.06.019 20833076

[B16] Lane PH Steffes MW Mauer SM Estimation of glomerular volume: a comparison of four methods Kidney Int 1992 41 4 1085 1089 10.1038/ki.1992.165 1513090

[B17] Chen HM Liu ZH Zeng CH Li SJ Wang QW Li LS Podocyte lesions in patients with obesity-related glomerulopathy Am J Kidney Dis 2006 48 5 772 779 10.1053/j.ajkd.2006.07.025 17059996

[B18] Gellman DD Pirani CL Soothill JF Muehrcke RC Kark RM Diabetic nephropathy: a clinical and pathologic study based on renal biopsies Medicine (Baltimore) 1959 38 321 367 13827229

[B19] Weight loss helps type 2 diabetes patients Dis Manag Advis 2008 14 10 11 12 19025166

[B20] Ramachandran A Ma RC Snehalatha C Diabetes in Asia Lancet 2010 375 9712 408 418 10.1016/S0140-6736(09)60937-5 19875164

[B21] Wu Y Overweight and obesity in China BMJ 2006 333 7564 362 363 10.1136/bmj.333.7564.362 16916811 PMC1550451

[B22] Maric C Hall JE Obesity, metabolic syndrome and diabetic nephropathy Contrib Nephrol 2011 170 28 35 21659755 10.1159/000324941 PMC3177237

[B23] Meltzer AA Everhart JE Unintentional weight loss in the United States Am J Epidemiol 1995 142 10 1039 1046 7485049 10.1093/oxfordjournals.aje.a117557

[B24] Shen WW Chen HM Chen H Xu F Li LS Liu ZH Obesity-related glomerulopathy: body mass index and proteinuria Clin J Am Soc Nephrol 2010 5 8 1401 1409 10.2215/CJN.01370210 20498244 PMC2924407

[B25] Saiki A Nagayama D Ohhira M Endoh K Ohtsuka M Koide N Oyama T Miyashita Y Shirai K Effect of weight loss using formula diet on renal function in obese patients with diabetic nephropathy Int J Obes (Lond) 2005 29 9 1115 1120 10.1038/sj.ijo.0803009 15925953

[B26] Andreotti F Rio T Lavorgna A Body fat and cardiovascular risk: understanding the obesity paradox Eur Heart J 2009 30 7 752 754 19251724 10.1093/eurheartj/ehp081

[B27] Zoccali C The obesity epidemics in ESRD: from wasting to waist? Nephrol Dial Transplant 2009 24 2 376 380 18952700 10.1093/ndt/gfn589

[B28] Wang Y Medicine 2010 Houst, co: People’s Medical Publish

[B29] Chen HM Li SJ Chen HP Wang QW Li LS Liu ZH Obesity-related glomerulopathy in China: a case series of 90 patients Am J Kidney Dis 2008 52 1 58 65 10.1053/j.ajkd.2008.02.303 18423814

