# Supplementary material for: The relationship between obesity and diabetic nephropathy in China
Source: BMC Nephrol. 2013 Mar 25;14:69. doi: 10.1186/1471-2369-14-69 (PMC3614546; doi:10.1186/1471-2369-14-69)
Supplement: Additional file 1: Table S1 — Epidemiological characterisstics of subjects with diabetic nephropathy. NOTE. Values were expressed as expressed as mean ± SD and categorical data expressed as number (%). Abbreviation: BMI, body mass index. To convert serum creatinine in μmol/L to mg/dL, multiply by 0.0113; creatinine clearance rate in mL/s/1.73 m2 to mL/min/1.73 m2, multiply by 60.0. The listed P values are based on student’s test or Chi-square test. *P < 0.05 and **P < 0.01versus lean group. ‡P < 0.01 versus overweight group. [file 1471-2369-14-69-S1.docx]

**Supplemental Table 1. Epidemiological characteristics of subjects with diabetic nephropathy.**

|  | All DN | Analyzed DN | ***P* value** |
| --- | --- | --- | --- |
|  |  |  |  |
| No. of patients | 757 | 264 |  |
| BMI (kg/m^2^) | 25.9 ± 3.05 | 25.5±3.39 | 0.454 |
| Age (years) | 52.6 ± 9.54 | 53.1 ± 9.06 | 0.622 |
| Male sex (%) | 466（61.5%） | 154（58.3%） | 0.380 |
| Known duration of diabetes (months) | 107 ± 67.0 | 111 ± 73.7 | 0.249 |
| Known duration of proteinuria (months) | 23.2 ± 35.6 | 27.7 ± 41.7 | 0.326 |
| Fasting glucose (mg/ dL) | 128± 45.2 | 124 ± 44.8 | 0.132 |
| HbA1c (%) | 6.80 ± 1.46 | 6.66 ± 1.33 | 0.272 |
| Mean blood pressure (mmHg) | 105 ± 13.4 | 108 ± 14.2 | 0.470 |
| Proteinuria (g/24h) | 2.96 ± 3.04 | 3.09 ± 2.32 | 0.569 |
| Serum albumin (g/dL) | 3.62 ± 0.77 | 3.45 ± 0.74 | 0.251 |
| Serum creatinine (mg/dL) | 2.13 ± 1.86 | 2.02 ±2.02 | 0.847 |
| Ccr (mL/min/1.73m^2^) | 94.3 ± 42.9 | 97.8 ± 53.4 | 0.219 |
| Uric acid (μmol/L) | 373 ± 94.0 | 400 ± 108 | 0.147 |
| Hemoglobin (g/dL) | 10.8 ± 2.2 | 11.2 ± 2.6 | 0.216 |

NOTE. Values were expressed as expressed as mean ± SD and categorical data expressed as number (%).Abbreviation: BMI, body mass index. To convert serum creatinine in μmol/L to mg/dL, multiply by 0.0113; creatinine clearance rate in mL/s/1.73m^2^ to mL/min/1.73m^2^, multiply by 60.0. The listed *P* values are based on student’s test or Chi-square test. ^*^ *P* < 0.05 and ^**^ *P* < 0.01versus lean group. ^‡^ *P* < 0.01 versus overweight group.
